# Translational genomics in personalized medicine – scientific challenges *en route* to clinical practice

**DOI:** 10.1186/1877-6566-6-2

**Published:** 2012-06-19

**Authors:** Marta Garcia Martinez de Lecea, Michael Rossbach

**Affiliations:** Genome Institute of Singapore, 60 Biopolis Street, Singapore, 138672 Singapore; Office of Scientific Affairs, Genome Institute of Singapore, 60 Biopolis Street, Singapore, 138672 Singapore; Office of Business Development, Genome Institute of Singapore, 60 Biopolis Street, Singapore, 138672 Singapore

**Keywords:** Biomarkers, Bioinformatics, Cancer, Genomics, Next-generation sequencing, Personalized medicine, Economics, Ethics

## Abstract

**Background:**

In the area of omics and translational bio(medical)sciences, there is an increasing need to integrate, normalize, analyze, store and protect genomics data. Large datasets and scientific knowledge are rationally combined into valuable clinical information that ultimately will benefit human healthcare and are *en route* to clinical practice. Data from biomarker discovery and Next Generation Sequencing (NGS) are very valuable and will combine in comprehensive analyses to stratify medicine and guide therapy planning and ultimately benefit patients. However, the combination into useful and applicable information and knowledge is not trivial.

**NGS in personalized medicine:**

Personalized medicine generally promises to result in both higher quality in treatment for individual patients and in lower costs in health care since patients will be offered only such therapies that are more effective for them and treatments that will not be safe or effective will be avoided. Recent advancements in biomedical and genomic sciences have paved the way to translate such research into clinical practice and health policies. However, the move towards greater personalization of medicine also comes along with challenges in the development of novel diagnostic and therapeutic tools in a complex framework that assumes that the use of genomic information is part of a translational continuum, which spans from basic to clinical research, preclinical and clinical trials, to policy research and the analysis of health and economic outcomes. The use of next-generation genomic technologies to improve the quality of life and efficiency of healthcare delivered to patients has become a mainstay theme in the field as benefits derived from such approaches include reducing a patient’s need to go through ineffective therapies, lowering side- and off-target effects of drugs, prescribing prophylactic therapies before acute exacerbations, and reducing expenditures.

**Economic challenges:**

As such, personalized medicine promises to increase the quality of clinical care and, in some cases, to decrease health care costs. Besides the scientific challenges, there are several economic hurdles. For instance, healthcare providers need to know, whether the approach of personalized healthcare is affordable and worth the expenses. In addition, the economic rationale of personalized healthcare includes not only the reduction of the high expense of hospitalizations, the predictive diagnostics that will help to reduce cost through prevention or the increased efficacy of personalized therapies needs to offset prices of drugs. There are also several factors that influence payer adoption, coverage and reimbursement; the strength of evidence drives payers‘ decisions about coverage and reimbursement, varies widely depending on the personalized healthcare technology applied and regulation and cost-effectiveness seem to be increasingly associated with reimbursement, which is strongly influenced by professional society guidelines. In general, we see the following main obstacles to the advancement of personalized medicine: (i) the scientific challenges (a poor understanding of molecular mechanisms or a lack of molecular markers associated with some diseases, for example), (ii) the economic challenges (poorly aligned incentives), and (iii) operational issues in public healthcare systems. The operational issues can often be largely resolved within a particular stakeholder group, but correcting the incentive structure and modifying the relationships between stakeholders is more complex.

**En route to clinical practice:**

This article focuses on the scientific difficulties that remain to translate genomics technologies into clinical practice and reviews recent technological advances in genomics and the challenges and potential benefits of translating this knowledge into clinical practice, with a particular focus on their applications in oncology.

**Electronic supplementary material:**

The online version of this article (doi:10.1186/1877-6566-6-2) contains supplementary material, which is available to authorized users.

## Background

Next-generation sequencing (NGS) is revolutionizing the way of genomic-scale biological research, and its effects are starting to translate into the clinic. In this sense, the comprehensive sequencing of the genome, epigenome and transcriptomes of cancers and corresponding “normal” (germ-line) DNA are heralding the start of personalized medical genomics. The promise of truly personalized medicine and individualized treatments is certainly appealing, however, several issues must be resolved before NGS will be adopted as a routine application in the clinic and for patients. Among such issues are the lack of integrated data, the need for relevant methodologies and research frameworks, and the clinical complexities of genome-based diagnostics and therapeutics. In personalized genomic medicines, pharmacogenomics involves the study of analyzing the individual variations in drug response; pharmacogenetics involves the analysis of genetic variation in drug metabolism. Contemporary studies increasingly involve pathway and network analyses that include both pharmacokinetics, i.e. factors that influence the concentration of a drug reaching its target, and pharmacodynamics, i.e. factors associated with the drug target, as well as genome-wide association studies (GWAS) approaches. In regard to the technology foundations, high-throughput tools for nucleic acid characterization provide the means to conduct comprehensive analyses of all alterations in a patient’s genome. Together with the rapid developments in human genomics, these disciplines move towards a system-based translational interface in pathophysiology, bringing genomic studies into the clinic. This further leads to the identification of new targets and novel biomarkers for both diagnostics and therapeutics. To translate biomarker- and NGS-technology-based research into clinical practice, diagnostics is key. The goal is a tailored approach to treatment based on the molecular analysis of genes, proteins, and metabolites. Yet, although this approach has generated much excitement, only a few personalized-medicine tests have achieved high levels of clinical adoption and are mostly in the field of oncology. To better understand the challenges to the development and acceptance of personalized medicine it is important to analyze the current technologies and complement this information with a (micro)economic view on the broad stakeholder issues in “P4 medicine”, which is *predictive, personalized, preventive*, and *participatory* medicine.

This personalized medicine will be fueled by systems approaches to disease, emerging technologies and novel analytical tools, including novel tools in bioinformatics, data analysis and integration of algorithm development.

## Next-generation sequencing technologies

Today, sequencers can analyze hundreds of millions of DNA fragments per run, compared with 1 to 384 DNA fragments in traditional sequencing methods. Even the read-lengths obtained with the new sequencing methods are significantly shorter that with the traditional Sanger sequencing method (up to 250 nucleotides vs. > 1000 nucleotides), the volume of sequence produced is much higher. Currently, several major sequencing platforms are available for next-generation sequencing (NGS), i.e. the 454 Life Sciences ([http://www.454lifesciences.com]) (Genome Sequencer™, GS junior) ([454 Life Sciences], 2011), the SOLiD™ technology from Life Technologies (5500 series Genetic Analysis Systems) ([http://www.lifetechnologies.com]) and the sequencing by synthesis (SBS) technology (Genome Analyzer™, HiSeq 1000/2000, MiSeq, HiScanSQ) from Illumina ([http://www.illumina.com]). All these technologies share the same basic principles: The Desoxyribonucleic Acid (DNA) of interest is ligated to two adaptor sequences and immobilized on beads. Subsequently, Polymerase Chain Reaction (PCR) amplifies the signal from individual DNA fragments via the adapter sequences and the amplified signal, from “beads” in the SOLiD system or from “clusters” in the Genome Analyzer, is analyzed using labeled nucleotides. Digital images are captured and analyzed to determine the individual nucleotides in the captured DNA.

Very recently, new players have emerged in the race toward the US $1,000 genome, among them Pacific Biosciences’ single molecule real time (SMRT™) sequencing technology and the semiconductor technology offered in the Ion Torrent PGM™ system ([http://www.iontorrent.com]). In the DNA sequencing process, the PacBio *RS* system uses advanced collection optics to record light pulses emitted as a byproduct of nucleotide incorporation; algorithms translate these signals into an A, C, G or T base call ([http://www.pacificbio.com]). The Personal Genome Machine from Ion Torrent ([http://www.iontorrent.com]) uses a massively parallel array of proprietary semiconductor sensors to perform direct real-time measurement of the hydrogen ions produced during DNA replication. A high-density array of wells on the Ion Semiconductor Chips provides millions of individual reactors while integrated fluidics allow reagents to flow over the sensor array. The combination of fluidics, micromachining, and semiconductor technology enables the direct translation of genetic information to sequence information ([http://www.iontorrent.com]).

With such new technologies in the field of genomics, it is anticipated, that the US $1,000 genome will become reality no later that 2013 (Wolinsky 2007; Thomas 2011). RainDance’s Sequence Enrichment application focuses on the targeted sequencing of the human genome using the RainStorm™ microdroplet-based technology for high-resolution analysis of genetic variations between individuals. The RainStorm technology utilizes a library of PCR primers enabling the amplification of hundreds to thousands of genomic loci in a single tube avoiding limitations of traditional multiplex hybridization and amplification technologies. RainDance’s application for sequence enrichment improves target enrichment uniformity and reduces the selection bias typically associated with targeted sequencing ([http://www.raindancetech.com]).

In regards to analyzing cancer genomes in the context of personalized medicine, the biggest driver to adopt NGS technologies has been the rapid decrease in the costs of sequencing per gigabase. This reduced cost has allowed for the analysis of cancer genomes at a single-nucleotide resolution. Nowadays, NGS technologies are used to identify all types of cancer genomic rearrangements and mutations within a single experiment, including large-scale chromosomal aberrations, single-nucleotide variations, structural variations (SV), small and large insertions, deletions or translocations or copy number variations (CNV). To obtain a complete determination of all kind of aberrations in a patient’s cancer genome, genomic data can be integrated with epigenomic and transcriptomic data. In terms of epigenomics, changes in DNA methylation, histone modifications or nucleosome positioning are detected and transcriptomic analyses focus on fusion genes, allele-specific expression or expressed mutations of aberrant gene expression patterns. However, such integrated analyses require huge resources for data generation, integration and interpretation. Individual tumors may accumulate more than 10.000 mutations and rearrangements, with only a small percentage of those are likely to be oncogenic. Therefore, large-scale sequencing projects enable to distinguish between those mutations that contribute to tumorigenesis, progression or metastasis, and those that accumulate in the tumor but do not contribute to disease.

Such projects further expand the repertoire of druggable targets and enable advancements in the development of personalized genomics and individual treatment plans. In cancer genomics, germ-line tumor DNA from each individual patient needs to be sequenced to understand the genomic heterogeneity at the single-nucleotide level. This is also interesting in regards to studying genomic heterogeneity in populations and germ-line variations and to understand how these may affect response to treatment, the tolerance to side- and off-target effects, and drug metabolism.

Furthermore, NGS technologies are of great importance in view of cataloguing normal human genetic variations like Single Nucleotide Polymorphisms (SNP) or Structural Variants (SV). Several large-scale projects intend to identify such variations, e.g. the 1000 genomes project or the database of genomic variants (DGV), to enable pathological genetic changes to be identified and to understand their disease relevance. Also, it is important to study genomes and analyze genetic variations from a variety of human populations and individuals from different ethnic backgrounds. This will ensure that personalized medicine is not restricted to those populations within which most genomics research has been conducted to date.

The extraction of knowledge from integrated genomic data, the comprehensive analysis and its transfer into healthcare IT systems is fundamental and requires not only state-of-the-art genomic but also IT infrastructures.

## The clinical potential of translational genomics

### Biomarkers

Biomarkers are simple molecules describing changes and alterations both in physiological and structural aspects of cells and tissues, at different levels from genes to whole tissues. The National Health Institute has defined biomarkers as "A characteristic that is objectively measured and evaluated as an indicator of normal biologic processes, pathogenic processes, or pharmacologic responses to a therapeutic intervention." Among the principal appliances of biomarkers to personalized medicine, the important ones are their specificity and sensitivity. This represents a highly attractive characteristic in revealing a disease predisposition or state, and in predicting a drug behavior or response to treatment. Additionally, biomarkers are very suitable for drug targeting, compound development, or for clinical trials evaluation. In regards to drug development, the utilization of biomarkers represents a powerful tool at preclinical and early stages, particularly for treatment design.

**Figure 1:**
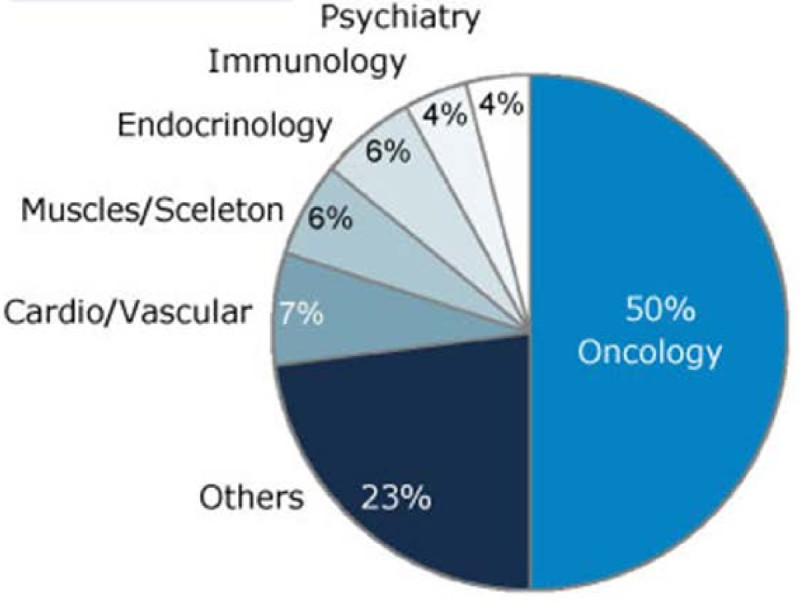
**Clinical studies with biomarkers.** More than 50% of all clinical studies that involve biomarkers are in performed in the field oncology (http://www.clinicaltrials.gov, 30.000 studies from 1970 until February 2011, The Boston Consulting Group analysis).

**Figure 2:**
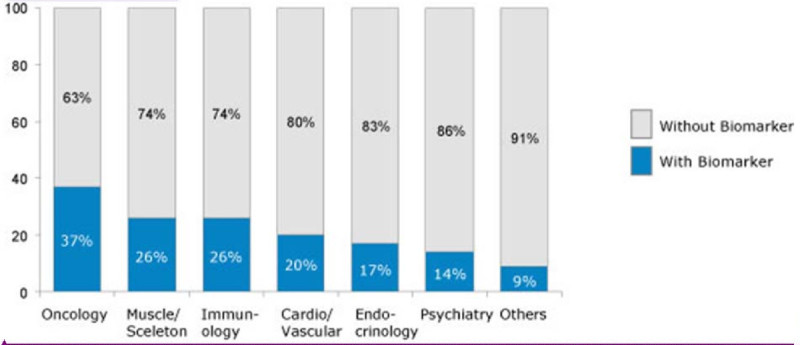
**More than 1/3 of all clinical studies in oncology include biomarkers.** The graphic illustrates the involvement of biomarkers in clinical studies, sorted by therapeutic area, with more than 1/3 of all clinical studies in oncology involving biomarkers (http://www.clinicaltrials.gov, 30.000 studies from 1970 until February 2011, The Boston Consulting Group analysis).

Today, several clinical studies include biomarkers with more than 50% of all clinical studies that include biomarkers being performed in the field of oncology (Figure 1); more than 1/3 of all clinical studies in cancer research include biomarkers (Figure 2).

Genetic tests to predict the susceptibility of an individual to a certain disease or to identify specific genetic mutations even though the individual remains unaffected represents one of the most derivations of personalized medicine. Also, genetic tests and – as a very new field – virtual disease-modeling can predict the behavior of a patient to certain drugs and will lead towards enhances therapeutics and patient treatment strategies.

Tumor biomarkers are crucial tools for diagnosis, and can be used to monitor disease progression, predict prognosis and detect recurrence of disease. They can be of particular value when it comes to predicting the survival outcome of patients and in determining the appropriate treatment plan and course of therapeutic intervention. NGS technologies contribute to the development of such biomarkers since the large-scale sequencing projects of various tumor types allow the identification of new candidate biomarkers, including non-coding Ribonucleic Acids (ncRNAs), microRNAs, aberrantly expressed genes, epigenetic markers or copy-number variations. Several currently available biomarkers lack sensitivity of the specificity to screen for all kind of tumors. As such, the discovery of markers with higher specificity for different types of cancer will assist in personalizing a patient’s treatment plan. The ability to monitor a treatment response or to detect tumor recurrence at early stages is key to ensuring that patients receive optimal therapy regimens. Genome sequencing of primary tumors is performed to analyze structural variations and changes such as translocations and leads towards the identification of truly personalized biomarkers. Once identified by sequencing, structural variants can be detected in the circulating DNA in plasma samples by PCR (Leary et al., 2010). Such methods, requiring only a few milliliters of blood, will potentially allow the development of very sensitive personalized biomarkers to monitor disease progression, recurrence and the success of the selected therapy.

### Personalized medicine

Patients with the same type of a tumor may respond in many different ways to treatment, given the heterogeneity of cancer. The sequencing of tumor cells will identify potential therapeutic targets or pathways that are silenced or activated in an individual tumor and, consequently, can be used to identify, which drugs and compounds are most likely to succeed in an individual patient.

Since many cancer drugs come along with undesirable and severe side effects, personalized treatments intend to minimize such effects and may maximize the outcome for each patient and avoid therapies that will have none or only little effects on the tumor.

**Figure 3:**
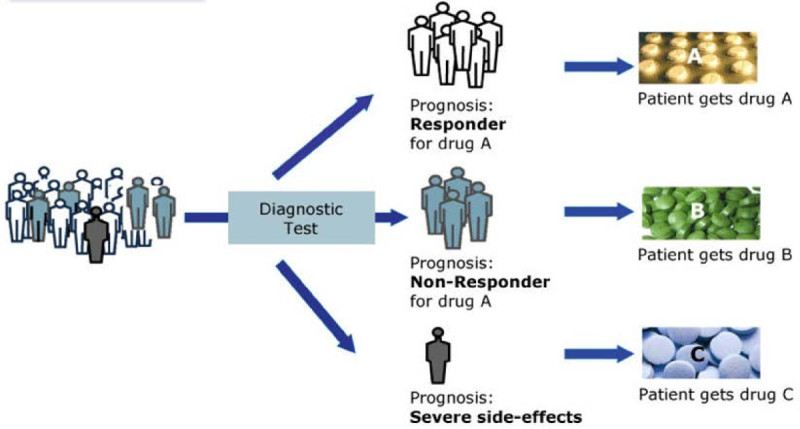
**Better patient treatments through advanced diagnostics and personalized medicine Diagnostic tests will guide the clinical decision-making to prescribe a specific drug, depending on the patient’s prognosis to be a responder or non-responder to a given medication (VFA Bio).**

This approach is not limited to cancer treatment and better patient treatment is achieved through novel and advanced diagnostic tests that will give a prognosis for an individual patient in terms of responder or non-responder to a certain therapy (Figure 3).

Thus, a truly personalized approach in genomic-based medicine will significantly enhance the quality of life of cancer patients. Examples for individualized treatments of cancer include HER2/neu-positive breast cancers that are treated with the monoclonal antibody trastuzumab (Herceptin™) and poly(ADP-ribose) polymerase (PARP-) inhibitors used in clinical trials to treat patients with BRCA1- or BRCA2-deficient breast cancers (Fong et al. 2009).

### Translating cancer genomic data into biomedical knowledge

Large-scale cancer genomic projects will identify and enumerate the frequency of every genetic element of interest that is altered in its structure in a cancer genome.

The statistical significance for such alterations is based on the frequency, which is an important filter to distinguish driver from passenger mutations. However, it is likely that mutations occurring at a very low frequency also contribute to specific types of cancer. In such cases, additional experimental evidence is important to identify low-frequency events as contributors to cancer initiation or progression (Chin and Gray 2008). Often, multiple genetic alterations contribute to the behavior of any specific cancer; thus, additional functional analyses are required to complement the genome annotation. A prominent example can be found in the *ras* oncogenes involved in human cancers. It has been demonstrated that the mutation status of *k-ras* dictates the response of tumors with EGFR mutations to treatment with EGFR inhibitors and that the complex relations of *braf*, *craf*, and *ras* in response to selective *braf* inhibitors (Heidorn et al., 2010; Poulikakos et al., 2010) confirm that further functional studies are necessary to exploit knowledge of somatic mutations. Another level of complexity is added due to the fact that genes not mutated in cancers may very well contribute to the survival and progression of tumors with other genetic aberrations. An example is the *parp1* gene, whose inactivation is synthetically lethal in breast and ovarian cancers that are deficient of BRCA1 or BRCA2 (Fong et al., 2009, Fong et al., 2010).

## The challenge: progressing genomic technologies into clinical practice

### Technologies and methods

Sequencing-based genomic technologies, which mainly employ traditional Sanger Sequencing technology (capillary electrophoresis), are not new to the clinical laboratory and range from infectious disease to cancer diagnostics. Like other molecular diagnostics, Sanger Sequencing enables the analysis of specific gene sequences for identifying a pathogenic agent, predicting or monitoring disease, selecting treatment options, and determining a treatment’s efficacy. Examples include the sequencing of the human immunodeficiency virus (HIV) to monitor the susceptibility or resistance to HIV antiviral medications, the sequencing of the cystic fibrosis (CF) gene or sequencing of the BRCA1 and BRCA2 genes in breast cancer. NGS-based technologies have a great potential to identify, classify and analyze disease sub-types, in particular in the elucidation of the contribution of rare, highly penetrant mutations to the genetic architecture of complex disorders, for instance in the monogenic form of morbid obesity and Type 2 diabetes. Such knowledge is very valuable in the delivery of personalized medicine

Also, for rare and orphan disease in general, NGS- and biomarker-based analyses can help to identify relevant loci in disorders and thus might stimulate drug development in this area.

In oncology, NGS-based systems will allow for the rapid sequencing of tumor genomes to direct cancer treatments, pathogen genomes to determine drug resistance, or cell-free DNA to identify chromosomal abnormalities in pregnancy, and offer undisputed advantages over Sanger Sequencing. The time involved to generate and analyze genomic data in a medical context represents one of the major challenges *en route* to apply NGS technologies as a standard tool in clinical practice. However, as technologies develop and progress, sequencing at a cost of US$ 1,000 might be possible in the near future. If genomic data feed clinical decision-support systems to evaluate the best possible therapeutic intervention, then these results should ideally be available within a short timeframe, i.e. rather within a few days than weeks. To achieve this, the steps performed during the analyses, which include library preparation, sequencing runs and the integrated data interpretation, must be fast, reliable and fully automated. The integrated and advanced analysis of genomic data comes along with the collection of such data; automatic analysis software for detecting somatic mutations is being developed and is tested for its sensitivity, specificity and accuracy. The challenge lies in assigning the biological significance of each somatic mutation and integrating each data type, *viz.* genomic, epigenomic or transcriptomic, to predict the biological impact. The biological understanding of the human genome is far from complete and although somatic mutations and aberrations may be detected, the information would not be informative to the clinicians if the genes affected have not been studied so far or if the pathways they are involved in are unknown.

Another challenge is the starting material required to generate the sequencing libraries. Samples with a limited amount of starting material, e.g. from core biopsies, are beyond the scope of the most thorough genomic analyses. For transcriptomic libraries, typically a depletion of the ribosomal RNA (rRNA) is required to allow the signal from expressed genes to be detected and such depletion does not work well with degraded RNA, affecting mostly formalin-fixed samples. Normal DNA can be derived from blood samples; however, gene expression and epigenomic variation differ significantly in different tissues. Thus, adjacent healthy tissue is required to determine the default and “normal” status of the gene expression, epigenome and trancriptome. Depending on the size and location of a specific tumor, this could be very challenging.

### Predictive, preventive, personalized and participatory medicine

As a result to all current efforts to translate the concepts of personalized healthcare into the clinic, personalized medicine will become participatory and this implies patient decisions about their own health. As a prerequisite, such a new paradigm requires powerful tools to securely handle significant amounts of personal information with the approach introduced as P4 medicine that is *predictive, preventive, personalized* and *participatory*. The main benefits from P4 medicine include enabling the design of drugs based on proteins and RNAs molecules (Rossbach, 2010) associated with genes and diseases, developing therapies that are highly targeted to specific diseases in order to maximize the effects of therapeutic interventions while minimizing side-effects or damaging healthy tissues. Avoiding trial and error phases through better and truly personalized drugs, using genetic profiles to identify the best possible drug and therapy for a given patient and reducing adverse effects are instances of personalized medicine goals, will benefit not only patients, but the public healthcare systems in general.

**Figure 4:**
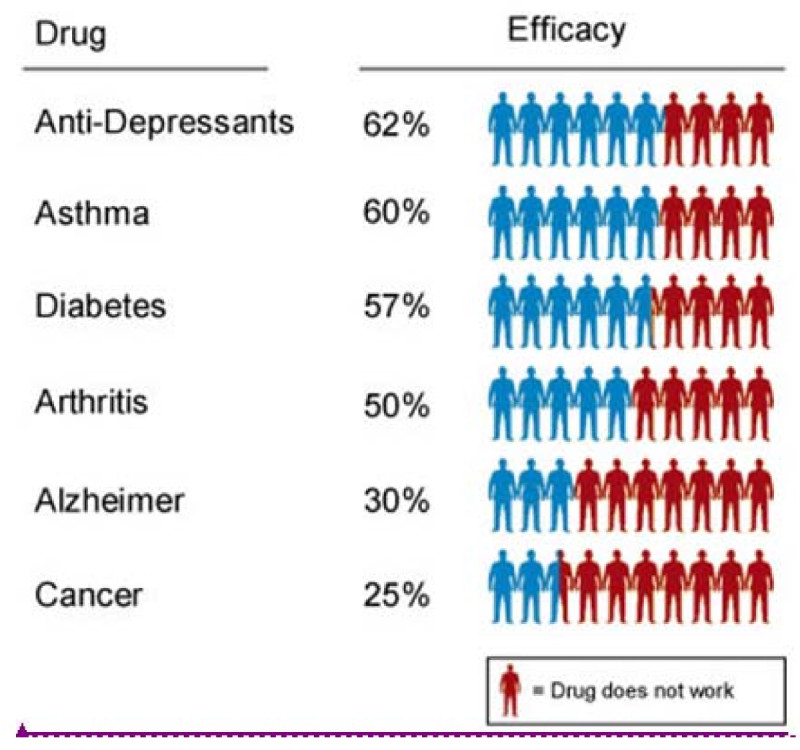
**The Challenge: Selection of the suitable drug In conventional medical approaches, valuable drugs go to unsusceptible patients.** (Spear et al., 2001).

The P4 medical approach will also help to select the right drug for the right patient at the right time, avoiding that valuable drugs are prescribed to unsusceptible patients and preventing potentially harmful side-effects (Figure 4).

In this regard, novel genome-based diagnostic technologies offer a significant progress in medical practice in comparison with the current prevention methods; a combination of genetic knowledge and clinical studies is expected to impart a significant advance toward preventive medicine and subsequent prospective medicine.

### Genomic literacy: education and counseling

Large-scale sequencing project in cancer medicine are crucial since cancer is a highly complex genetic disease and the hereditary causative mutation of a combination of mutations is only know for a very small number of familial cancers. As such, NGS approaches translated into clinical practice have the potential to unravel some of this genetic complexity. Such knowledge can provide genetic counselors with information that is required to feed clinical decision-support systems when selecting the best possible treatment plan and therapeutic intervention for an individual patient. Also, such data would provide the information necessary to inform family members of potential risks of a disease and to ensure that these family members have access to the most appropriate screening programs and preventions. The knowledge gained also leads to the development of more preventive rather than curative therapies.

### Economic aspects

Along with the discussions of translating genomic knowledge into clinical practice come considerations of the economic value, namely careful assessments of benefits, risks and costs (Collins et al., 2003; Haga and Burke 2004). With regards to the cost-effectiveness of different genomic screening strategies, more evidence is needed since most analyses so far do not take the impact of testing on quality of life into account. Assessments should consider the complexity for screening for genomic aberrations, including the development of reliable algorithms for integrated data analysis. Incorporating subgroups or family members of affected patients, should also be considered. To assess the value of personalized medicine, it is important to define approaches that consider both diagnostic tests and the treatment with drugs. Genome-based diagnostic tests should elucidate whether the detected mutations are relevant to the given phenotype and if this phenotype can be linked to clinical predictions. From a market’s perspective, the diagnostic market represents a large and fast growing opportunity for NGS, much greater than the currently addressed life science research market, which is mostly driven by biopharmaceutical research and development (R&D) or government funding. However, the diagnostics market also entails much larger regulatory and reimbursement hurdles. Thus, to successfully translate NGS-based systems into clinical practice, it will be crucial to develop valuable, user-friendly, reliable, and cost-effective diagnostic tests. To facilitate routine testing in clinical practice and laboratories, the market is demanding cost effective and simple-to-perform tests that have cleared the many regulatory hurdles. Also, automation is playing a key role in the development of diagnostic tests that are easier and less expensive to operate. Given the re-emergence of infectious threats, *viz.* multidrug-resistant TB, new strains of HIV or H1N1, infectious disease testing represents a large portion of the current market for molecular diagnostics. Here, pharmacogenomics will likely be the most immediate new opportunity in the field and many pharmaceutical companies invest significantly in pharmacogenomics in anticipation of shaving years off the drug discovery and approval process, bringing potentially lucrative drugs to market much sooner (Constance, 2010).

In regards to personalized cancer diagnostics, the molecular oncology diagnostics sector is expected to grow at a compound annual growth rate (CAGR) of approx. 18% per annum; through 2015, the molecular diagnostics market will grow at double-digit pace, achieving an overall 14% CAGR to meet increasing demand for personalized medicine. Key areas of growth include not only oncology an infectious diseases, but also genetic testing and biobanking with a wide variety of drugs in late preclinical and early clinical trials being targeted to disease-specific gene and protein defects that will require co-approval of diagnostic and therapeutic products by the regulatory authorities.

### Ethical aspects

Ethical issues come along with the advancements and progression of translational genomics and the debate is mostly about discrimination, confidentiality and data security or informed consent. Unlike most diagnostic tests, whole genome sequencing allows the identification of an individual and related family members. Thus, concerns regarding data security and privacy are raised and access to genomic patient data must be restricted to the use in clinical practice.

As discussed, there are both substantial challenges and benefits in translating genetic results about disease susceptibilities or genomic data into clinical practice. Studies on individual (cancer) genomes and genetic susceptibility produce large volumes of biomedical data on a wide range of phenotypes. The impact of genetic data can extend well beyond clinical utility as there may be emotional, rather than practical benefits and risks of genetic risk information if provided to a patient; the emotional meaning may be more relevant for very large amounts of genetic information and information on susceptibility and risk, rather than prediction/diagnosis and people may react very differently to this kind of data, based on personality/coping strategy and the context of the information. Thus, patients should be encouraged to consider the range of results, how they might cope, and the range of possible actions/reactions before performing NGS-based diagnostic tests. As genetic studies on susceptibility to disease proliferate, it s necessary to demand replication and understanding of population attributable risks of significant findings. As patients and consumers seek out genetic profiling and will ask for such profiles, physicians need to understand the clinical validity and limitations of the data for making health-related decisions and should be trained to interpret the complex results of personal genomic sequencing data.

We need well-designed studies about how patients/consumers are affected by genetic data, especially large amounts of data, in order to understand the risk and benefits.

Another aspect is the equality of access in relation to further delivery of personalized treatments since novel drugs in this particular field are very expensive and ubiquitous reimbursement schemes are not in place in most countries yet.

## Conclusions

At present, little is known about the use, preferences, and value of genome-based approaches in personalized medicine (Khoury et al., 2006). Further research is required to overcome the challenges associated with the translation of genomic knowledge into clinical decision-making in terms of patient data, testing procedures, algorithm development and the use of such information in therapy planning.

As the NGS technologies are advancing, it is very likely that these technologies will be translated into the clinical labs and hospitals and not only guide the treatment of patients who have failed to respond to first-line therapies. With these advancements, a better understanding of human cancer genomes comes along. The comprehensive analysis of such genomes in healthy and diseased samples will generate comprehensive genomic, epigenomic and transcriptomic data present in diverse human tumors and the structural and functional analysis of these data will allow for the development of novel biomarker-based diagnostics, innovative therapeutics and will guide clinical trials. The main focus has been on known cancer pathways so far, but the full application of these approaches will lead to the identification of novel pathways and players that maintain the malignant state, thus providing the framework to develop novel diagnostics and therapeutics. However, collecting accurate clinical information on tumor samples remains a challenging task and often samples are associated with incomplete annotation. Challenges associated with bringing NGS-based data into the clinic are not only scientific, like accounting for the genetic heterogeneity within tumors, but are also operational and legal, in terms of patient consent and data protection. In the future, NGS technologies will further advance, thus allowing the analysis of patterns of genetic heterogeneity on the single-cell level. Integrative analyses will then be able to determine how the heterogeneity affects tumor initiation, progression, recurrence or a patient’s response to treatment. Another step is to make genomic data available in useful formats that can be applied to clinical decision-support, selecting from treatment options and to plan therapeutic interventions. The ability to applying advanced genomic technologies prospectively in patients, together with integrated functional analyses, will provide a deeper understanding of tumor biology, and will also facilitate the selection of the best available treatment option for each and every individual patient.

## Authors’ original submitted files for images

Below are the links to the authors’ original submitted files for images. Authors’ original file for figure 1 Authors’ original file for figure 2 Authors’ original file for figure 3 Authors’ original file for figure 4
