# Supplementary figures and images for: Translational genomics in personalized medicine – scientific challenges en route to clinical practice
Source: Hugo J. 2012 Jun 19;6(1):2. doi: 10.1186/1877-6566-6-2 (PMC4685154; doi:10.1186/1877-6566-6-2)

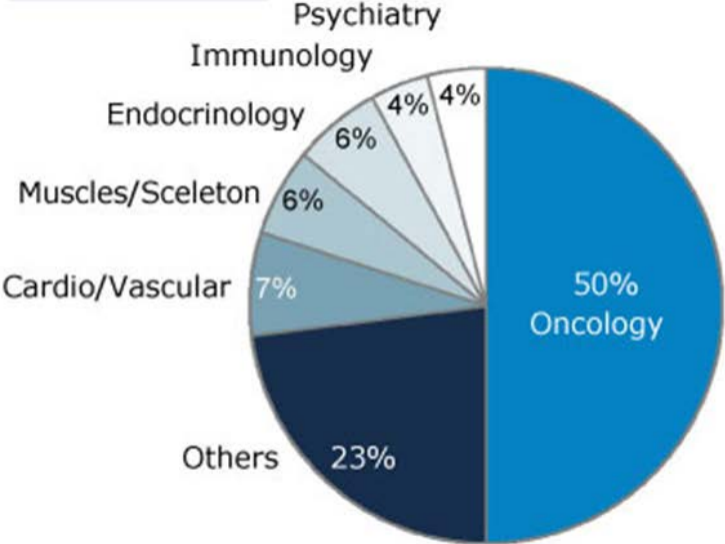

Supplement: Supplementary file 1 — Authors’ original file for figure 1 [file 11568_2011_2_MOESM1_ESM.pdf]

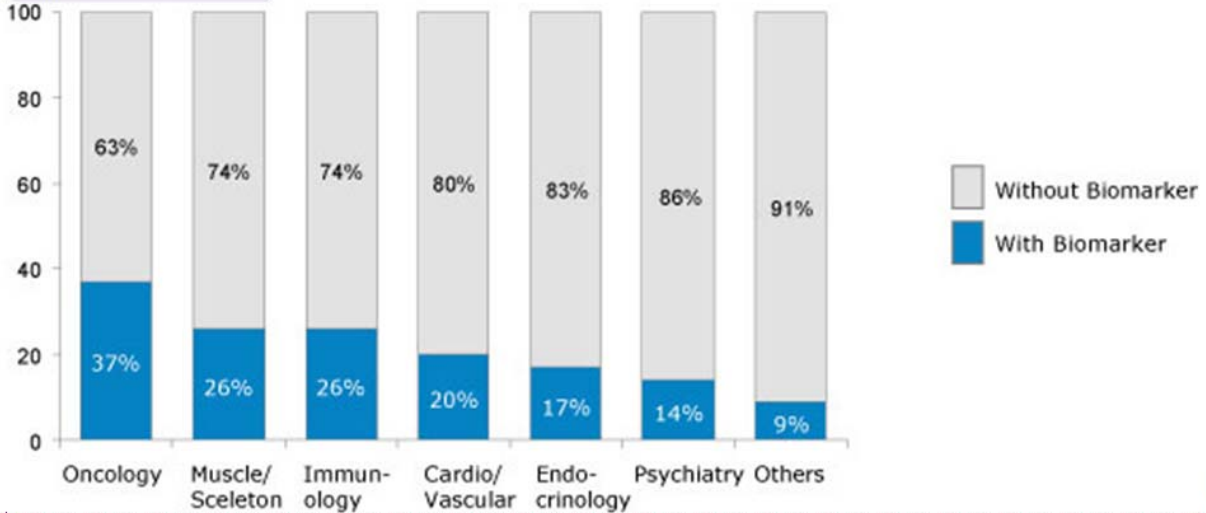

Supplement: Supplementary file 2 — Authors’ original file for figure 2 [file 11568_2011_2_MOESM2_ESM.pdf]

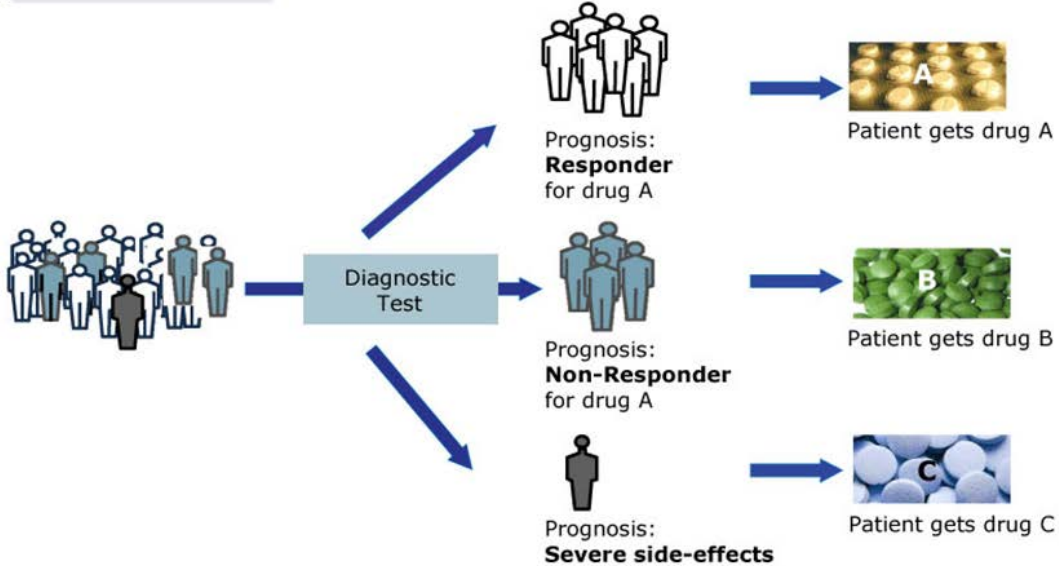

Supplement: Supplementary file 3 — Authors’ original file for figure 3 [file 11568_2011_2_MOESM3_ESM.pdf]
