# Supplementary material for: Translational genomics in personalized medicine – scientific challenges en route to clinical practice
Source: Hugo J. 2012 Jun 19;6(1):2. doi: 10.1186/1877-6566-6-2 (PMC4685154; doi:10.1186/1877-6566-6-2)

Drug	Efficacy
Anti-Depressants	62%
Asthma	60%
Diabetes	57%
Arthritis	50%
Alzheimer	30%
Cancer	25%

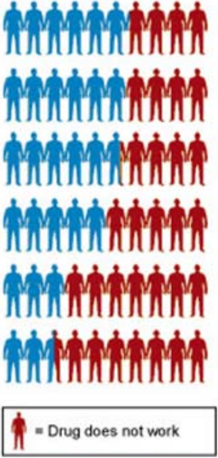

Supplement: Supplementary file 4 — Authors’ original file for figure 4 [file 11568_2011_2_MOESM4_ESM.pdf]
